# Deep learning ensemble 2D CNN approach towards the detection of lung cancer

**DOI:** 10.1038/s41598-023-29656-z

**Published:** 2023-02-20

**Authors:** Asghar Ali Shah, Hafiz Abid Mahmood Malik, AbdulHafeez Muhammad, Abdullah Alourani, Zaeem Arif Butt

**Affiliations:** 1 Department of Computer Sciences, Bahria University, Islamabad, Pakistan; 2 Faculty of Computer Studies, Arab Open University Bahrain, A’ali, Bahrain; 3 Department of Computer Science and Information, College of Science in Zulfi, Majmaah University, Al-Majmaah, Saudi Arabia

**Keywords:** Biotechnology, Cancer, Computational biology and bioinformatics, Health care

## Abstract

In recent times, deep learning has emerged as a great resource to help research in medical sciences. A lot of work has been done with the help of computer science to expose and predict different diseases in human beings. This research uses the Deep Learning algorithm Convolutional Neural Network (CNN) to detect a Lung Nodule, which can be cancerous, from different CT Scan images given to the model. For this work, an Ensemble approach has been developed to address the issue of Lung Nodule Detection. Instead of using only one Deep Learning model, we combined the performance of two or more CNNs so they could perform and predict the outcome with more accuracy. The LUNA 16 Grand challenge dataset has been utilized, which is available online on their website. The dataset consists of a CT scan with annotations that better understand the data and information about each CT scan. Deep Learning works the same way our brain neurons work; therefore, deep learning is based on Artificial Neural Networks. An extensive CT scan dataset is collected to train the deep learning model. CNNs are prepared using the data set to classify cancerous and non-cancerous images. A set of training, validation, and testing datasets is developed, which is used by our Deep Ensemble 2D CNN. Deep Ensemble 2D CNN consists of three different CNNs with different layers, kernels, and pooling techniques. Our Deep Ensemble 2D CNN gave us a great result with 95% combined accuracy, which is higher than the baseline method.

## Introduction

Deep learning and machine learning algorithms provide state-of-the-art results in almost every field of life, including wireless sensor networks, order management systems, semantic segmentation, etc^1–3^. It hugely impacts bioinformatics, specifically cancer detection^4,5^. Cancer is a disease with the most death toll. It is the most dangerous disease ever known to humans. Cancer is still not curable as the people suffering from it come to know about it in the later stages. It is complicated to detect it at an early stage, and more cancer-related deaths are mostly lung cancer. Therefore, significant research has been conducted to develop a system that can detect lung cancer from CT scan images^6^. It is challenging to prevent cancer as it shows signs in the later stages where it is impossible to come out of it. So, people can only do a regular checkup every six months, especially those who drink and smoke. This study aims to develop a state-of-the-art system for the early detection of lung nodules using the latest proposed ensemble deep learning framework.

**Figure 1:**
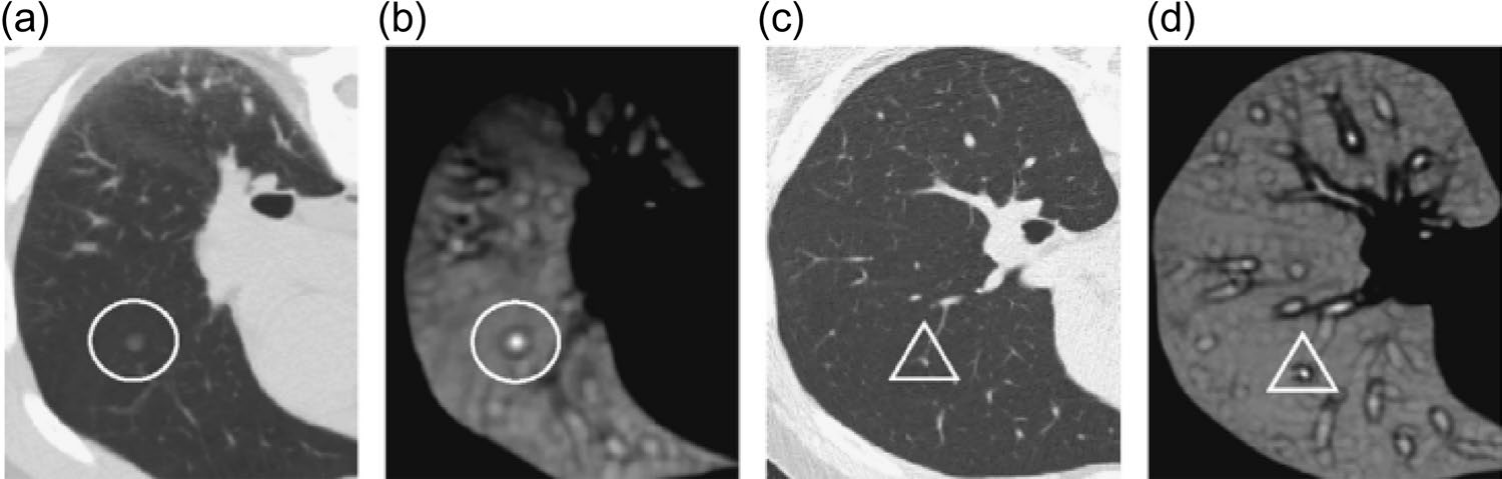
CT scan images show lung nodules with different locations and shapes in CT.

According to the latest report of the World Health Organization, death caused by Lung Cancer has moved from the top 9 to the top 6 in the list of diseases that cause the most significant number of deaths^7^. Lung Cancer has different types: small cell lung cancer and non-small cell Lung Cancer^8^. Figure 1 explains the CT Scan images used to detect the presence of a Lung Nodule, a cancer tumor. All tumors are not cancerous; the primary tumor types are Benign, Premalignant, and Malignant^6^.

In this research, we have used a supervised deep learning model CNN because we need to classify the result as cancerous or non-cancerous. The simplest definition of understanding deep learning is, It learns from examples. It works like our brain works to learn from examples. For the concerns mentioned above related to Lung Nodule detection for early diagnosis of Lung Cancer, an ensemble of 2D CNN approaches has been developed to detect Lung Nodules. The data set used in this research is LUNA 16 Grand Challenge. Medical Sciences is one of the industries becoming an active part of practicing different machine learning and deep learning-based computerized automated software to balance the workload. With high-performance computing coming into the big picture, deep learning is becoming an active part of the research industry. The most critical role any deep learning model can play is to increase the system's efficiency, quality, and diagnosis to detect certain diseases more accurately and way before time to improve treatments and clinical results. Medicine and Health care are witnessing more implications if these deep learning and machine learning-based systems increase the accuracy of prediction and detection of diseases. Cancer is one of the most important parts of clinical research now a day's due to its high death rate and fewer chances of cure. Early detection of different cancer types can help in reducing the number of deaths all over the world. For the concerns mentioned earlier related to Lung Nodule detection for early diagnosis of Lung Cancer, an ensemble of 2D CNN approach has been developed to detect Lung Nodules. The data set used in this research is LUNA 16 Grand Challenge.

An Ensemble approach has been developed to help detect Lung nodules because it is tough to differentiate between a Lung Nodule and a Lung Tissue. For this purpose, a more accurate model should be developed to distinguish between the Lung Nodule Candidate and the actual Lung Nodule. Primarily the main issue faced by any researcher is the acquisition of relevant annotations/labeled image data instead of the availability of image data. All Free-text reports based on radiologists' findings are stored in the format of the PACS system. So, converting all these reports into more appropriate and accurate labeling of data and structural results can be a daunting task and requires text-mining methods. These text-mining methods themselves are an essential field of study. Deep learning nowadays is also widely used with text mining. In this regard, developing a structured reporting system will benefit Machine and Deep Learning objectives. This development can lead to the improvement of radiologic findings, and the patient care CAD system can help radiologists take the responsibility of more than one doctor. The Lung Nodule detection process includes a detailed inspection of Nodule Candidates and True Nodules. Lung Nodule candidates consist of true and false nodules resembling true ones. So, a classification system should be developed to select true nodules among all possible candidate nodules. Two challenges need to be addressed with more attention to establishing such nodules to detect true nodules.

Non-Nodules are highlighted, and some nodules are ignored in the CT scan, which is the radiological heterogeneity. It can lead to increased difficulty in differentiating between nodules and non-nodules. Nodules are in different sizes and different shapes. Larger nodules have a better tendency to be detected by the system, whereas small nodules have fewer chances, adding more to the challenges. Different shapes of a nodule are another factor that needs to be addressed by the model.

## Related work

Many studies used deep learning and ensemble learning processes for classification problems^9^. The current CAD applications for Lung Cancer classifying lung nodules are very close to this paper's objective. Therefore, we researched the recently developed and state-of-the-art lung nodule classification techniques.

### 2D convolutional neural network

A two-dimensional CNN has been used to detect lung nodule presence in the CT scan. In 2D CNN, CNN only takes two dimensions. Around the image to get the main features and learn these features, CNN with a transfer learning approach was developed by Wangxia Zuo, Fuqiang Zhou, and Zuoxin Li^10^ with MultiResolution CNN and Knowledge Transfer for Candidate Classification in Lung Nodule Detection. Image-wise calculation with CNN and different depth layers applied for Lung Nodule classification on Luna 16 Data Set to improve the accuracy of Lung Nodule Detection with 0.9733 Accuracy. Sanchez and Bram van Ginneken^11^ developed CAD system for' pulmonary nodules using multi-view convolutional networks for False Positive Reduction. MultiView-KBC was developed for Lung Nodule Detection by Yutong Xie, Yong Xia, Jianpeng Zhang, Yang Song, Dagan Feng, Michael Fulham, and Weidong Cai^12^, which is based on Knowledge-based Collaborative Deep Learning for Benign-Malignant Lung Nodule Classification on Chest. Siddharth Bhatia, Yash Sinha, and Lavika Goel present a deep residual learning approach using CT Scan for cancer detection^13^. ResNet^14^ and UNet models are used for feature extraction in this method. Machine learning algorithms XGBoost and RF (Random forest used to classify cancerous images. The accuracy of this model was 84%. The research proposed by Muhammad Imran Faisal, Saba Bashir, Zain Sikandar Khan, and Farhan Hassan Khan uses machine learning and ensamble learning methods to predict lung cancer through early symptoms. This study use different machine learning algorithms, including MLP (multilayer perceptron)^15^, SVM (Support vector machine)^16^, Naïve Bayes, and Neural network for the classification of lung cancer. The dataset used for this study is extracted from UCI repository. The accuracy of the ensemble learning method for the proposed study was 90%^17^.

### 3D convolutional neural network

Same as 2D CNN, but in this 3-Dimensional CNN, CNN considers three dimensions while learning the features like x, y, and z. Two sides are considered at once, like x and y, y and z, and z and x. False-Positive Reduction in Lung Nodules Detection using Chest Radiographs by an Ensemble of CNN was developed by Chaofeng Li, Guoce Zhu, Xiaojun Wu, and Yuanquan Wang^18^. For false positive reduction on Chest Radiographs with a fivefold cross-validation Multilevel contextual Encoding to detect the variable size and nodule shapes developed by Qi Dou, Hao Chen, Lequan Yu, Jing Qin, and Pheng-Ann Heng^19^. An Architecture developed to reduce the number of False Positives achieved 87% sensitivity with four false positives/scans. Qing Wu and Wenbing Zhao proposed a novel approach to detecting Small Cell Lung Cancer, and they suggested the entropy degradation method (EDM) for detecting Small Cell Lung Cancer. Due to the data set limitations, they developed their novel neural network, which they referred to as (EDM). They used 12 data sets: 6 were healthy, and six were cancerous. Their approach gave 77.8% accurate results in detecting Small Cell Lung Cancer. Wasudeo Rahane, Himali Dalvi, Yamini Magar Anjali Kalane, and Satyajeet Jondhale^20^ used Machine Learning techniques to detect Lung Cancer with the help of Image Processing**.** Data were pre-processed with different image processing techniques so the machine learning algorithm could use it; a Support Vector Machine for the classification was used. Allison M Rossetto and Wenjin Zhou^21^ give an approach to Convolution Neural Networks (CNN) with the help of multiple pre-processing methods. Deep learning played a significant role in this research. The implementation of CNNs did the accuracy of automated labeling of the scans. The results showed consistently high accuracy and a low percentage of false positives.

As discussed in the above section, none of the studies use an ensemble learning approach of machine learning or deep learning to identify the lung nodule. The main issue of the previous results was the improper or small dataset for the detection taken from minimum subjects. The above section clearly shows that the accuracy of detection with more machine learning or deep learning algorithms is very low. The current proposed study is going to cover these loopholes of the studies.

## Proposed method

The previously presented studies had an issue with the ensemble learning approach. All the studies presented in the past did not use an ensemble learning approach of deep learning algorithms for lung cancer identification. As the ensemble learning approach gives the best average accuracies, this study will cover the loophole of the previous studies by using the ensemble learning approach on CNN algorithms using CT images taken from LUNA 16 dataset. A final solution Deep Ensemble 2D CNN is developed with the help of the Deep Learning Algorithm^22^ to detect Lung Nodules from CT Scan images. It is imperative to select which model should be used to detect Lung Cancer with the help of Deep Learning. Here, the Supervised Deep Learning Algorithm 2D CNN is used to detect lung nodules. This section explains every step of the Deep Ensemble 2D CNN model that performs to get the best results and help develop a CAD system for Lung Nodule Detection. The idea of this Ensemble CNN with different CNN blocks is to get the correct features, which are very important to classify a true nodule among candidate nodules. In the end, we have calculated Accuracy, Precision, and recall using the formula below^23,24^.

1 $$\mathrm{Accuracy}=\frac{TPV+TNV}{TPV+FPV+TNV+FNV}$$

2 $${\text{Precision }} = \frac{{{\text{TPV}}}}{{{\text{TPV}} + {\text{FPV}}}}$$

3 $${\text{Recall }} = \frac{{{\text{TPV}}}}{{{\text{TPV}} + {\text{FNV}}}}$$

In these equations, TPV is the true positive value, TVN is the True negative value, FPV is the False positive value, and FNV is the False-negative value^25,26^.

The step-by-step working of the model is explained as.

1. Access the dataset from Luna 16.
2. Data pre-processing (Data Balancing, Plotting, Data Augmentation, Feature extraction)
3. Splitting the dataset into training and testing data.
4. Applying Deep 2D Neural Network to the training and testing dataset.
5. Combine the prediction of Deep 2DNN.
6. Final Prediction of Lung cancer.

**Figure 2:**
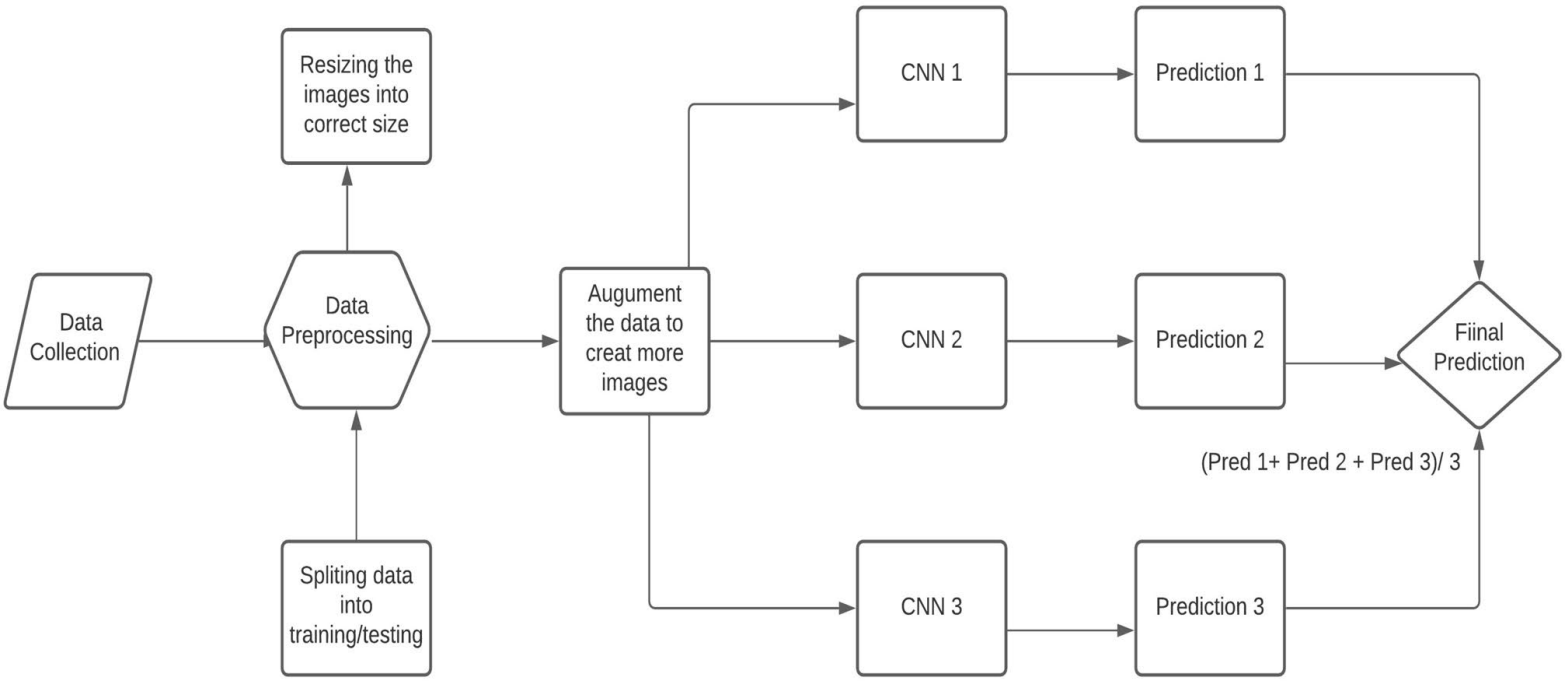
The architecture of the proposed methodology.

Figure 2 describes the research paradigm for the proposed model.

## Data collection

The crucial step in every research is the collection of data, as collecting the correct data helps get better results. The first step is organizing the enormous data set of CT Scan images. A Data set of CT Scan images were collected from LUNA 16 Data set which has helped to get the research completed^27^. It is essential to collect high-quality data so that the machines can understand the data easily. All CT Scan images are the same quality in showing the reports to any doctor. Images in the LUNA Data set were formatted as (.mhd) and (.raw) files. The .mhd files contained the header data, and the raw files had multidimensional image data. We used the SimpleITK python library to pre-process all these images to read all .mhd files.

## Data pre-processing

**Figure 3:**
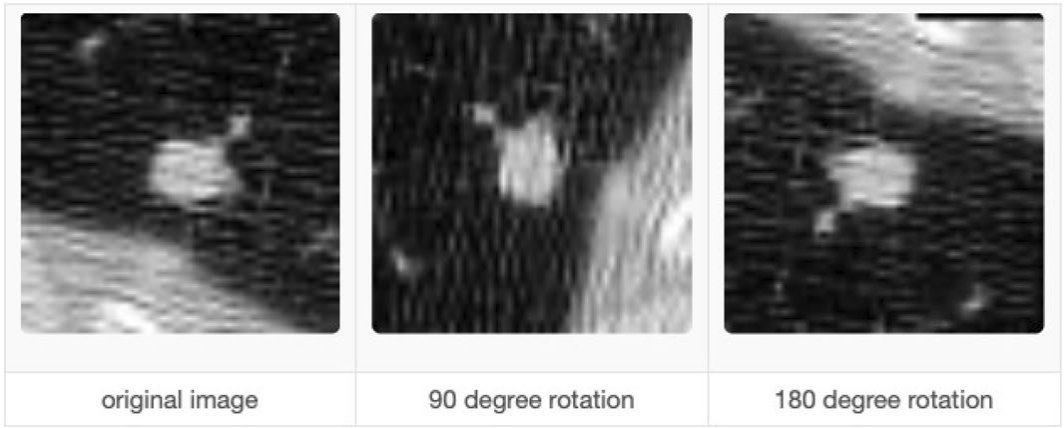
Cropped CT scan images.

The next step in the proposed solution is data pre-processing. It is a critical step in which data is converted into a more understandable form, making it very easy to understand and process by the machines^28,29^. It is the most vital step to transform data into the desired format so that devices can better understand it. All the CT scans in LUNA 16 Data set consisted of n 512 × 512 axial scans with 200 images in each CT scan. Only 1351 were positive nodules in these annotations, and all others were negative. There was an imbalance between the two classes, so we need to augment the data to overcome this issue. We can train the CNN model on all original pixels, increasing the computational load with training time. Instead, we decided to crop all images around the coordinates provided in the annotations. Figure 3 explains the dropped CT scan image from the dataset.

Furthermore, all the annotations provided in LUNA 16 Data set were in Cartesian coordinates. All these were converted to voxel coordinates. The image intensity in the dataset was defined in the Hounsfield scale. All these must be changed and rescaled for image processing purposes. All the images in the dataset belong to two classes which are positive and negative. Nodule Candidates with categories marked as 1 were positive and those with types marked as 0 were negative. Image Labels were created according to positive and negative. So finally, these label data can be used for training and testing.

**Figure 4:**
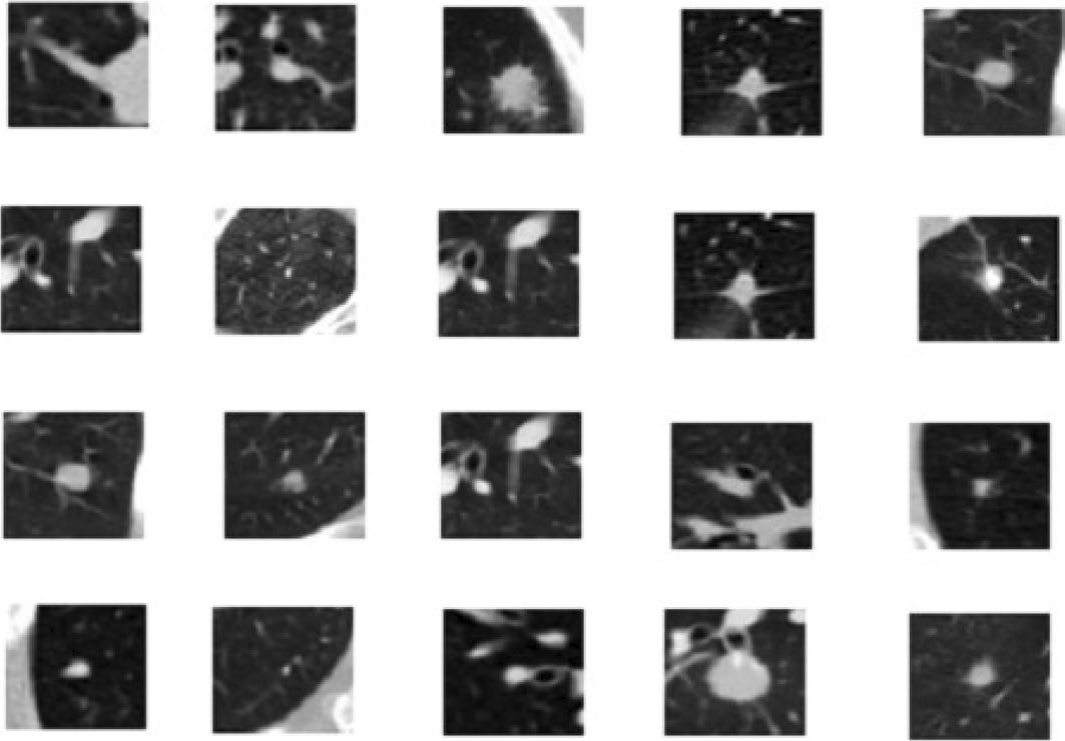
Input images to the proposed methodology.

It is usually in the format of Dicom images or MHD/Raw files. Before feeding data into any machine learning or deep learning model, it is crucial to converting the data into the required format so that machines can use it to understand and learn from it. Figure 4 shows the plotted image for the proposed system.

### Converting data into JPEG images

The next step is to convert all the pre-processed data into Jpeg format so that computers can understand it. Jpeg format is human readable, and humans can verify whether all the images are in the desired format, which can be seen and viewed easily in Jpeg format. Furthermore, the data was converted into small 50 × 50 images so that it would reduce the size of the data and it will consume less computing power. Hue data size consumes a lot of computing power, so to overcome this issue, images were reduced to 50 × 50.

### Data augmentation

It is imperative to augment the data when there is an imbalance issue. Manual data augmentation is done because data was not balanced. Data augmentation^30^ helps in this regard so that it rotates the images in all possible directions and makes a copy of them. This way, you can create more copies of the same data from a different angle, which helps solve the data imbalance issue. We also used Keras Image Data Generator for image pre-processing and data augmentation. Keras Image Augmentation will zoom in and out to learn more about image data shear range to flip an image. These are critical steps so that the data is possibly processed in all possible ways so machines can learn the data in each possible way.

### Split the data set into training and testing

The next important thing is splitting the data into testing and training or training and validation data. In this way, we can give machines the data to train and then provide the validation data to check the accuracy of our model. Reading the candidate's data from the CSV file and then splitting the cancerous and non-cancerous data so it can be correctly labeled. Making separate folders of cancer and non-cancer files is essential so that machines can learn what these files are and train. Training data is the data the artificial neural network and CNN will understand so they can learn more about the data and learn from it. It is a significant step to split the data so some portion of the data can be used for training. The next important thing is to give the test data to the artificial neural network and CNNs so the results can be generated and detect Lung Cancer form the CT Scan images can be done with the test data. Test Data is the actual data on which the algorithm's accuracy will be checked. If the result's accuracy is as required, then the results will be noted. If the results are not up to the mark, then some changes will be made to the layers in artificial neural networks and CNN to get more accurate results.

## Deep ensemble 2D convolutional neural network

**Figure 5:**
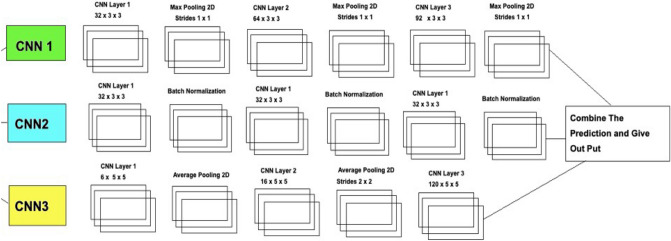
Deep ensemble 2D CNN architecture.

Figure 5 explains the different layers of the CNN Model. A final solution Deep Ensemble 2D CNN is developed with the help of the Deep Learning Algorithm to detect Lung Nodules from CT Scan images. It is essential to select which model should be used to detect Lung Cancer with the help of Deep Learning. This section explains every step our Deep Ensemble 2D CNN model will perform to get the best results and help develop a CAD system for Lung Nodule Detection. The idea of this Ensemble CNN with different CNN blocks is to get the correct features, which are very important to classify a true nodule among candidate nodules.

A Deep Ensemble 2D CNN Architecture was designed for an effective Lung Nodule Detection CAD system. A total of 3 2D CNNs have been designed and developed with different layers and pooling techniques. Each CNN in Deep Ensemble 2D CNN architecture has a different number of feature maps kernels with Max Pooling, Average Pooling, and Batch Normalization. Convolutional Layers in CNN Architecture do the feature extraction work. Each kernel convolves on the input and extracts the main features, which will help make output features later used for learning.

Keeping that in mind, we designed a Deep CNN model with more depth layers with a different number of feature maps, which will help extract true nodules among nodule candidates. The first layer in this Deep Ensemble, 2D CNN architecture, has 32 feature maps and 6 in the third CNN to learn the features of nodules with 3 × 3 and 5 × 5 kernel sizes. As the layers go deeper, we increase the number of feature maps with the same kernel size. As the neural network grows with more layers, more memory blocks are created to store the information, which helps to decide the nodule. Each CNN in this Deep Ensemble 2D CNN has a different number of layers and kernels.

**Figure 6:**
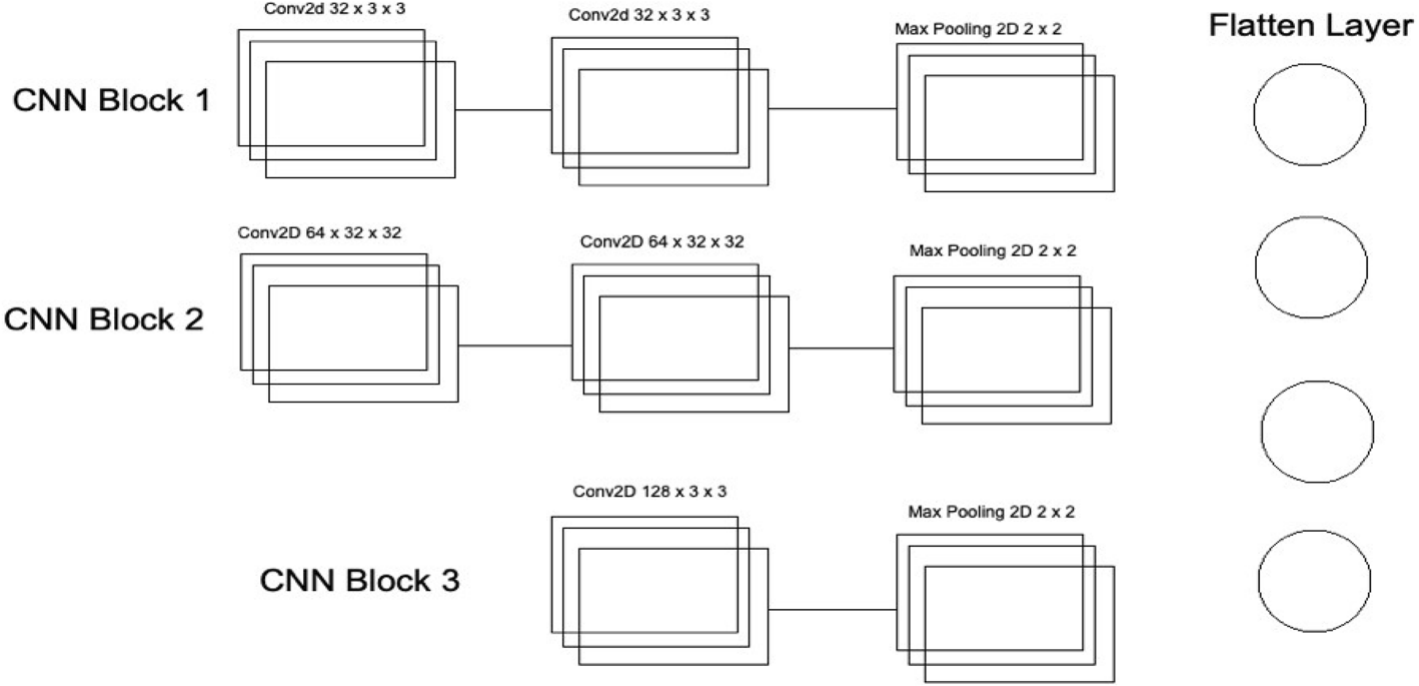
Convolutional neural network one architecture.

Furthermore, in CNN, Maxpooling^31^ is used to get the maximum value from the pooling layer filter. In the 2nd CNN, batch normalization is used, and in the third CNN, Average Pooling is utilized to get the average of all values. Furthermore, more depth layers were added to increase the accuracy and tuning of the architecture to overcome the over-fitting issues. More layers were introduced into this architecture to increase the efficiency of this model. This Deep Ensemble 2D CNN will help get more accurate modular features and minimize the false positives in the true nodules. In the end, the predictions of all three CNN will be combined to make a more accurate model. Using these predictions final confusion matrix was developed, which gave good results. Figure 6 illustrates the CNN architecture.

As mentioned in Deep Ensemble 2D CNN, this architecture is developed by developing and combining three different CNNs. This section explains the architecture of each CNN with several layers of each CNN. In CNN1 Architecture, three blocks of CNN are developed with a different number of layers and feature maps. The first CNN block has the 1st input layer of CNN, which uses a 3 × 3 kernel with 32 feature maps. In the first layer, RELU^32^ is used as an activation function. The input size is given in the first layer, and we have used the same image size, 50 × 50, and RGB channels as 3. Moving into the further hidden layers, in this first block of CNN, the next CNN layer has the same 32 feature maps with the same kernel size of 3 × 3. At the end of this 1st block, a Max Pooling 2D function will sub-sample the data. Our Max pooling filter size is 2 × 2, which will convolve on the data extracted by the feature maps, and it will use a 2 × 2 filter and get the maximum value from the data. This first CNN block is essential to get the features of the nodule and non-nodules. It will extract the main features that help to distinguish between the nodule candidate and the true nodule. Moving into the 2nd of CNN, the number of feature maps increased with 64 feature maps and kept the kernel size same to 3 × 3. The activation function is the same as the above layers, RELU, and the next CNN layer has the same number of feature maps and kernel size. Moving forward toward the Max Pooling layer in this 2nd block, there is the same Max Pooling as above, which is 2 × 2. In the last and third block of CNN, only one layer of CNN has 128 feature maps with the same kernel size, which is 3 × 3, and the Max Pooling layer is the same as above, which has a 2 × 2 size. In this 3rd block of CNN, we have a dropout rate of 0.1, meaning 10% of the neurons will be dropped in this layer to increase accuracy and avoid over and under-fitting issues.

**Figure 7:**
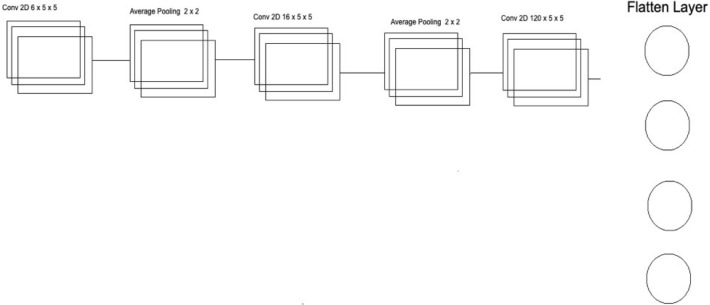
Convolutional neural network two architecture.

After the above CNN blocks, a Flatter layer will convert our CNN model into a one-dimensional layer, converting it into a pure ANN form. Then dense layers are added to make the architecture into a complete ANN form. In this layer, there is a dropout rate of 20. In the last layer, we have output dim as one because we need to predict only one result as nodule or non-nodule. Sigmoid is used as the activation function because we need binary output, not categorical, so Sigmoid is the best choice to predict the binary result^33^. Figure 7 illustrates the conversion of the CNN model array to flatten the layer.

The second CNN in Deep Ensemble 2D CNN Architecture is different in structure as a different number of layers and batch normalization is used instead of Max Pooling to sub-sample the data. In the very first layer of CNN, we used 5 × 5 size of kernels with six feature maps only instead of 3 × 3 because we tried to develop a different CNN as compared to the very first so we could know which feature map size could help get a more accurate result.

With strides of 1 × 1, the kernel filter will move one by one, and for activation, we have used RELU like the first CNN model. The exact size of 50 × 50 is used with three RGB channels for input shape. After the first layer, Average Pooling is utilized instead of Max Pooling. Average pooling works the same way as Max Pooling, but the calculation differs. In Max Pooling, we get the maximum value, and in Average Pooling, an average of data is calculated inside the feature kernel used to subsample the data. This Average Pooling uses a 2 × 2 size of kernel and strides of 1 to move the filter one by one. After the Average Pooling layer, there are some more hidden layers of CNN. The second Layer of CNN has 16 Feature maps with the same filter size of 5 × 5, keeping the stride the same to move one by one.

**Figure 8:**
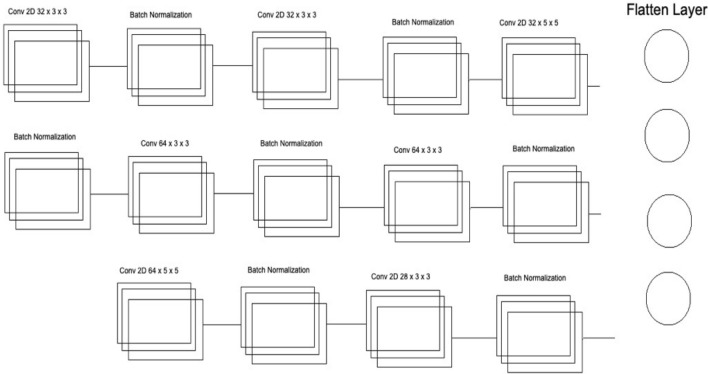
Convolutional neural network three architecture.

After this layer, there is another layer of Average Pooling. In this pooling layer, the same filter size 2 × 2 is there, but this time there is the strides of 2, which means our filter will move two steps instead of the traditional one-step movement. We need to get the features in every possible way and help our network to get the components in every possible way and learn from them. What features can it get by moving only one step, what features will it get by moving two steps each time, and how much will it help to understand the data better. In the last and third layers of CNN, we have used the same kernel size, which is 5 × 5 with 120 feature maps, and keeping the strides to 1. After this last layer, we have a flattened layer that will convert the CNN layers into one-dimensional ANN Architecture. After that, the traditional ANN is used to learn from CNN and classify the data. In the last layer, the Sigmoid activation function is utilized. Our results are binary, as we need to predict only nodules and non-nodule. If there is a need to predict more than two for any categorical data, SoftMax is a good option, as explained in Fig. 8.

Several layers and feature maps have been used in the last and third CNN Models of this Deep Ensemble 2D CNN Architecture. This CNN model uses three layers with 32 feature maps and a kernel size of 3 × 3. In the first layer, the input shape of the data is the same as the image size, which is 50 × 50, and the activation function used is RELU. 2 Layers of CNN have a 3 × 3 filter size, and the third layer has a 5 × 5 size. The dropout rate in this set of CNN layers is set to 0.4 after three layers of CNN, meaning 40% of neurons will be dropped. In this architecture of CNN, no average or max pooling is used. Instead, batch normalization has been used to increase the learning rate of the mode. In the hidden layers of this CNN model, three layers of CNN with 64 feature maps and 3 × 3 kernel size have been used. In the last layer of this CNN block, a 5 × 5 kernel size is used. After this block of CNN, the dropout rate is added to 0.4, which means 40% of the neurons will be dropped. Moving forwards in the third section of this CNN model, there is one layer of CNN with feature maps of 128 and kernel size of 3 × 3. After filtering this last layer, we have a flattened layer, which will convert the CNN layers into one-dimensional ANN Architecture. Later, the traditional ANN is used to learn from the CNN and classify the data.

Our Deep Ensemble 2D CNN used RELU as the activation function. Rectified Linear Units (RELUs) are a well-known and mostly used activation function in our proposed CNN model. A study from Krizhevsky et al.^34^ showed that RELUs enable the network to train several times faster than using the units in deep CNN. RELU is used for Input Layers and other multi-hidden layers in our Deep Ensemble 2D CNN.

As mentioned earlier, we used the Sigmoid activation function in the last layer^35^. Our results are binary, as we need to predict only nodules and non-nodule. If there is a need to predict more than two for any categorical data, then SoftMax is a good choice. Nonlinear Activation Functions make it easy for the model to adapt or generalize with a different type of data and differentiate between the output. Our classification task is the binary classification between nodule and non-nodule, so Sigmoid is the best choice for binary classification.

**Figure 9:**
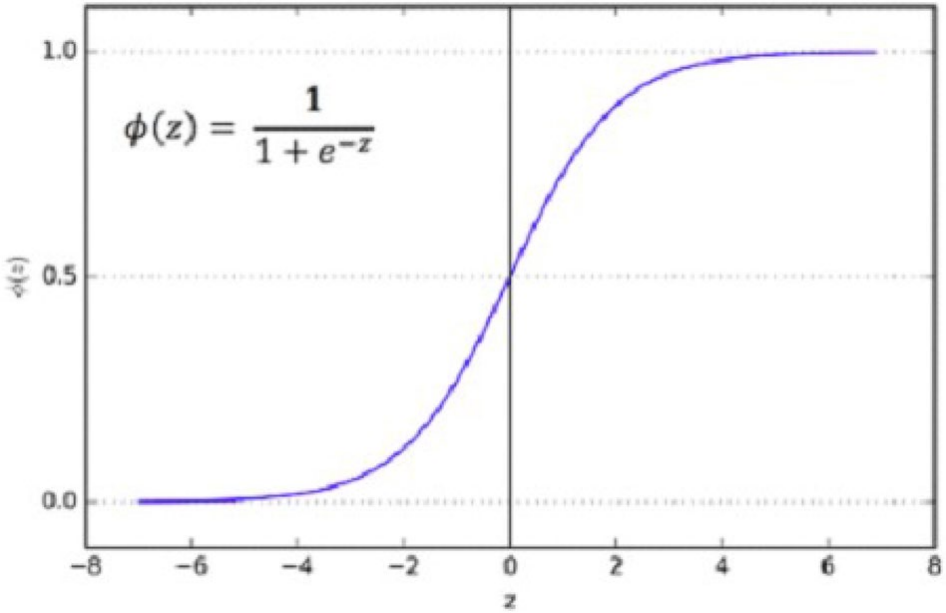
The Sigmoid curve at any two points.

Moreover, we mainly use the Sigmoid function because it exists between 0 and 1. Therefore, it is primarily used for tasks where we must predict the probability as an output. Since the probability of anything exists between 0 and 1, Sigmoid is the right choice. The function is differentiable. That means we can find the slope of the Sigmoid curve at any two points. Figure 9 shows the working of the sigmoid function.

## Experimental results and analysis

After pre-processing the data in the correct format, the very next important is to check the data on our Deep Ensemble 2D CNN Architecture. In this regard, the whole data was segmented into training and validation data. Both data segments have cancer and non-cancer lung nodule files, so the CNN Model can get to know both data types while training.

This section uses Deep Ensemble 2D CNN architecture and a validation split of 10%, which will help to use 90% of the data as training and the remaining as validation. It helps to make models train and test at the same time. With 70 epochs set in the model fit generator, it will iterate the dataset 70 times.

### Result of CNN1

**Figure 10:**
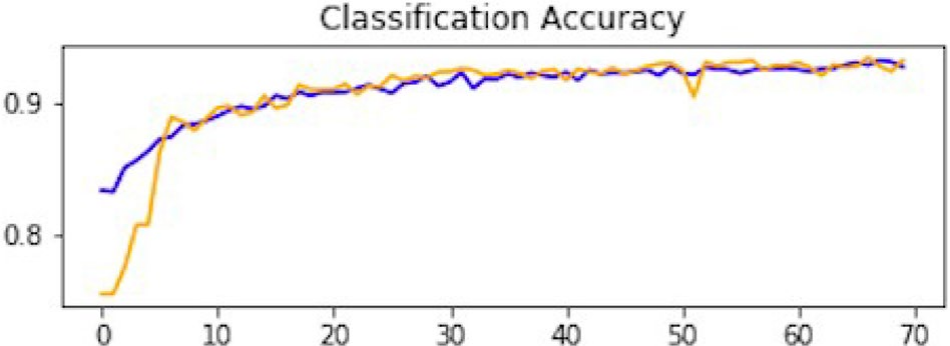
Accuracy curve of CNN1.

This section explains how each CNN has performed on the data. In the first CNN model, we first ran it on training and validation data. After the results, the test data was given to the model to predict the outcome of the CNN. The first iterative model of CNN provides an accuracy of 94.5%, which would be considered excellent results according to AUC accuracy values^36^. Figure 10 explains the results.

**Figure 11:**
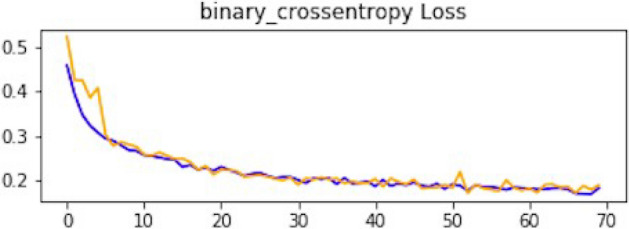
Loss curve of CNN1.

As mentioned, the model was compiled with 70 epochs^37^. Each epoch validation split divides 80% of the data into training and 20% of the data into validation. The training progress and epochs also show that the classification accuracy is increasing. At the same time, the loss of the model decreases rapidly at each iteration. The loss curve gives the result of 0.14 at the first iteration of CNN. The results of the Loss curve are described in Fig. 11.

Table 1 explains the training accuracy and loss for the first CNN model.

**Table 1:** Results of CNN1.

Training accuracy	0.9438
Testing accuracy	0.9453
Value loss	0.1454

**Figure 12:**
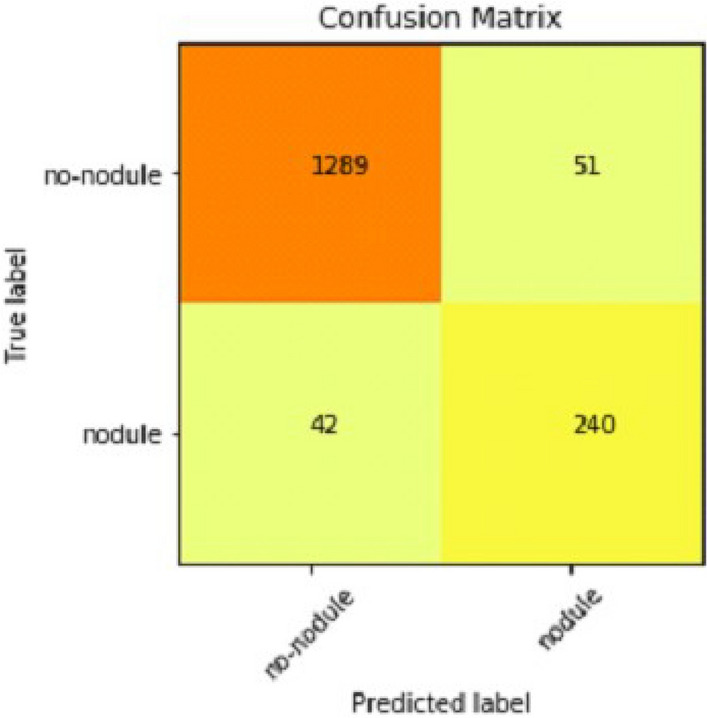
Confusion matrix of CNN1.

**Figure 13:**
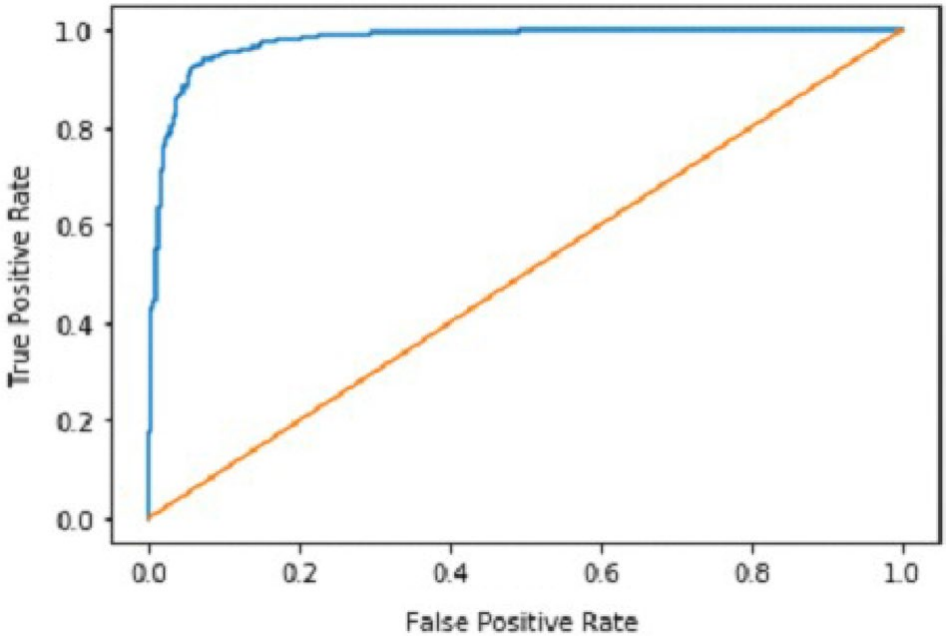
ROC curve of CNN1.

It gradually decreased as the model got more and more training in each epoch, and in the end, only a fraction of 0.1891 was recorded. According to the above results, accuracy is not enough to judge the model's performance. Later, we gave our model some data to predict the results and had around 1600 images to predict. After the prediction was made, the next step was to check the accuracy of the predictions, and for this purpose, we made a confusion matrix^38,39^. Below are the confusion matrix results for the data used for the first CNN layer. Here Nodules and Non-Nodules are the values of the detected and non-detected lung cancer images explained in Fig. 12. Figure 13 explains the ROC curve for the CNN model for the training dataset.

### Result of CNN2

**Figure 14:**
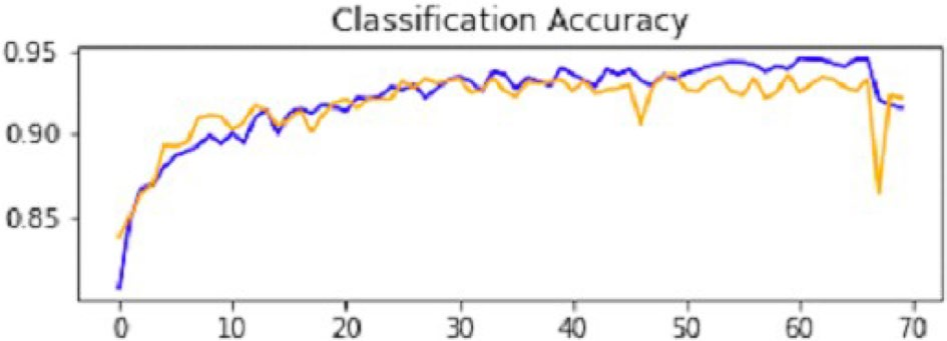
Classification accuracy of CNN2.

After the performance evaluation explanation of CNN1, moving forward in this section, it is explained how CNN2 has performed on the testing data. In the second CNN model, we first ran it on training and validation data. After getting the results, we gave this model the test data to predict the outcome of the CNN. The second model of CNN gave us some good results, which are stated below. The result also shows an accuracy of 0.93. The accuracy is gradually increasing from the first iteration to the last. Figure 14 explains the accuracy of CNN2.

**Figure 15:**
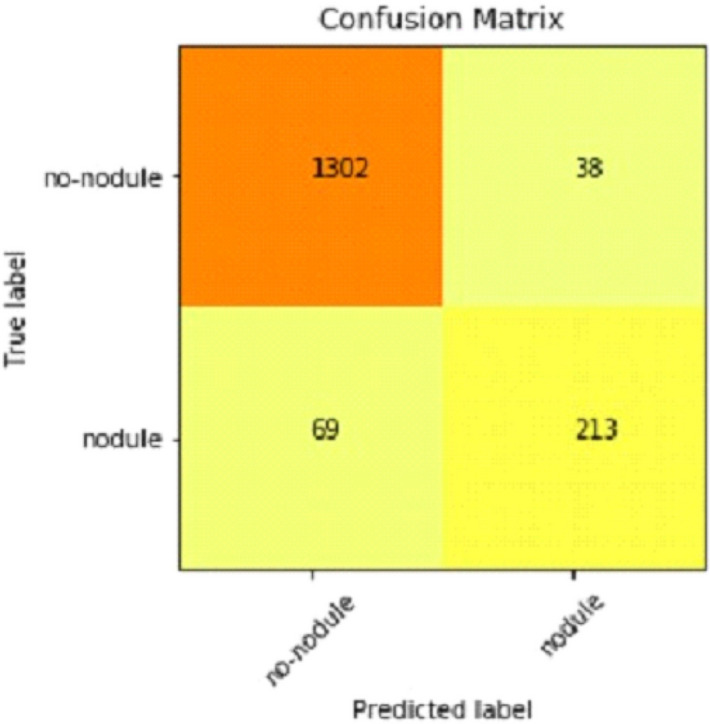
Confusion matrix of CNN2.

**Figure 16:**
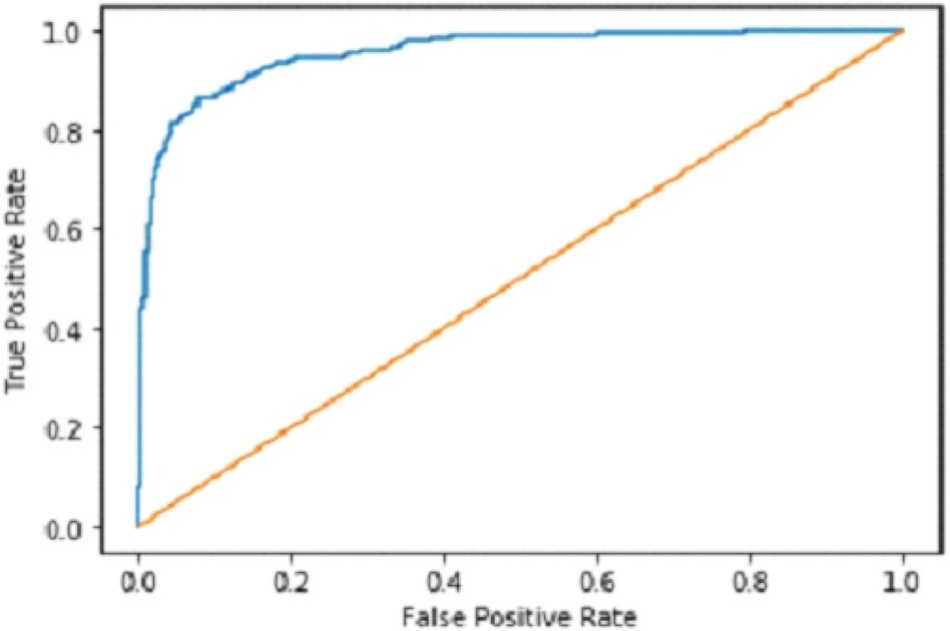
ROC curve of CNN2.

Table 2 shows that accuracy is insufficient to evaluate the model's performance. Afterward, we provided our model with information to forecast the outcomes and had roughly 1600 photos. The next step after making a prediction is to assess its accuracy, and a confusion matrix was created for this reason. The results of CCN2 for the testing images are explained in Fig. 15. The ROC curve for the testing dataset is presented in Fig. 16.

**Table 2:** Results of CNN2.

Training accuracy	0.9395
Testing accuracy	0.9391
Value loss	0.168

### Result of CNN3

**Figure 17:**
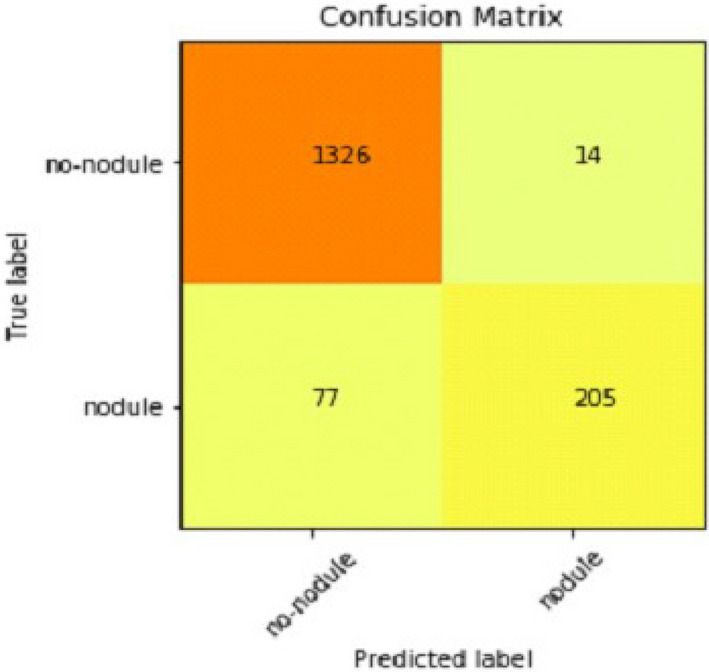
Confusion matrix of CNN 3.

**Figure 18:**
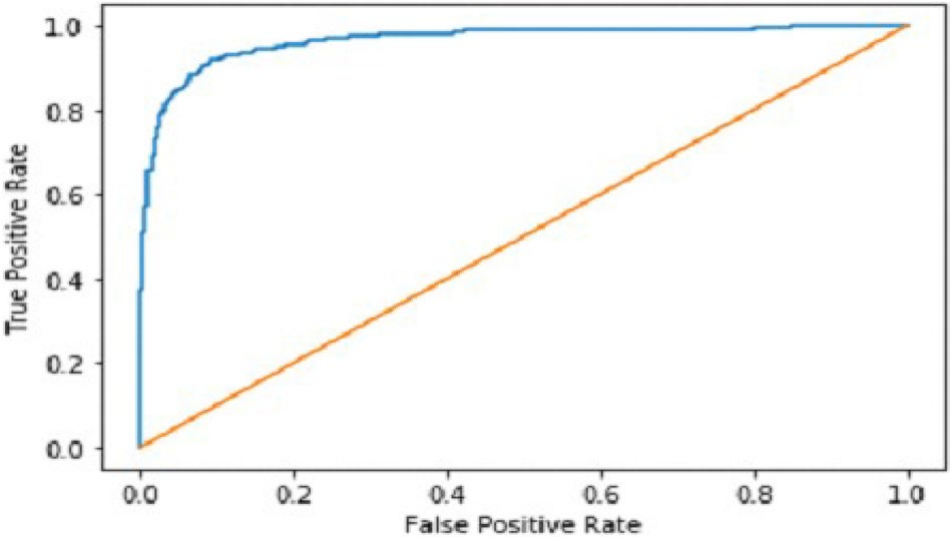
ROC curve of CNN 3.

We explain how CNN3 fared on the testing data in the sections that follow the discussion of CNN2's performance evaluation. We first tested the third CNN model using training and validation data. After the outcomes, we provided this model with test data to forecast how the CNN would turn out. As a result, the CNN third model produced some promising results, which we have included in Table 3. Figures 17 and 18 present the confusion Matrix and ROC curve for CNN3.

**Table 3:** Results of CNN 3.

Training accuracy	0.9297
Testing accuracy	0.9275
Value loss	0.1830

After the above results, accuracy is not enough to judge the model performance. After this, we gave our model some data to predict the results, and we had around 1600 images to predict. After the prediction was made, the next step was to check the accuracy of the predictions, and for this purpose, we made a confusion matrix. Below are the results.

### Combine results of all CNN (deep ensemble 2D CNN architecture)

After combining the prediction of all three CNNs, which we have designed especially for this Lung Nodule issue. We clearly can see from the confusion matrix that there is a difference in TP, TN, FP, and FN, which tells that combining all three CNN was an excellent choice to increase the accuracy and reduce the False Positives. The CNN architecture results are combined using the averaging method of deep ensemble learning^40^.

**Figure 19:**
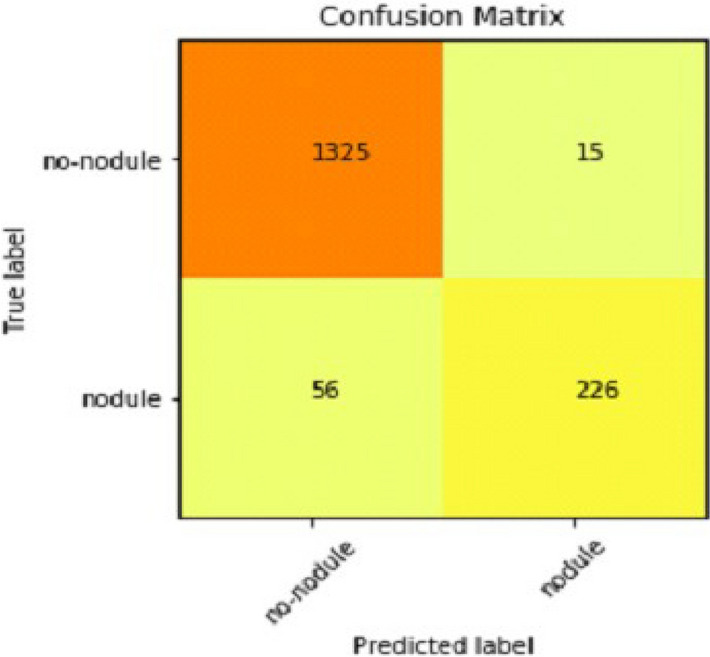
Confusion matrix of deep ensemble 2D CNN.

**Figure 20:**
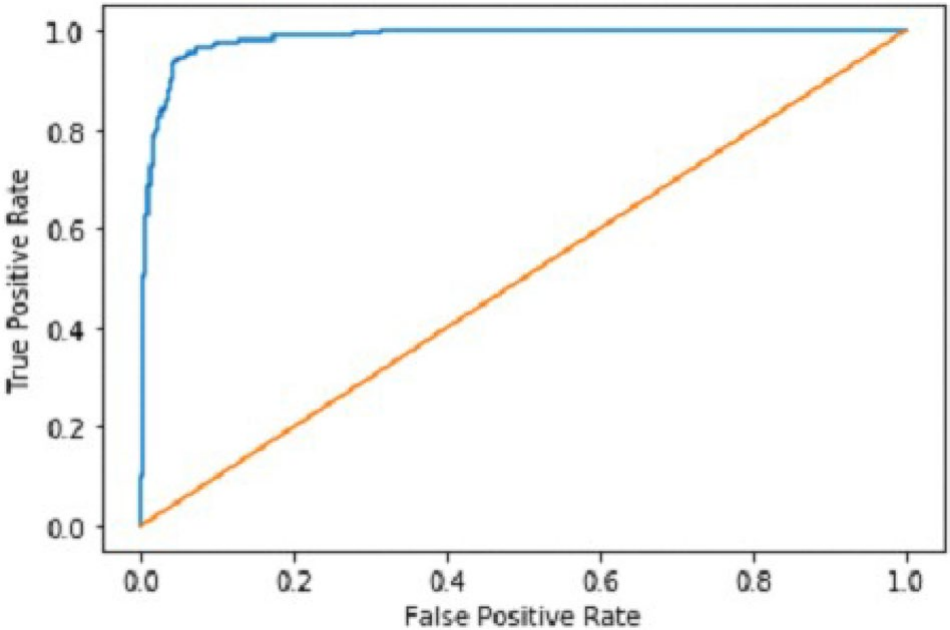
ROC curve of deep ensemble 2D CNN.

Our Deep Ensemble 2D CNN has three different CNNs, which achieve an accuracy of 90% and above. Our CNN1 attained an accuracy of 94.07%, CNN2 achieved an accuracy of 94.44%, and CNN3 attained an accuracy of 94.23%. Now we shall calculate the overall accuracy, precision, and recall of our Deep Ensemble 2D CNN from the confusion matrix. Table 4 illustrates the overall results of the CNN model. Figures 19 and 20 explain the combined confusion matrix and ROC curve results for the CNN model.

**Table 4:** Results of deep ensemble 2D CNN.

Accuracy	95%
Precision	0.93%
Recall	0.80%

### Comparison with other methodologies

Below we have stated the comparison between our proposed deep ensemble 2D CNN methodology and baseline methodology, which we considered in this approach to improve the accuracy and performance of the model. The comparison of the model with the base papers of the study is illustrated in Table 5.

**Table 5:** Comparison of the result with base papers.

Author	Methodology	Results
Sanchez and Bram van Ginneken^11^	Knowledge-based collaborative deep learning	94% Accuracy
Chaofeng Li, Guoce Zhu, Xiaojun Wu, and Yuanquan Wang^18^	Chest radiography with 5-fold cross-validation	87% Sensitivity
Siddharth Bhatia, Yash Sinha, and Lavika Goel^13^	Machine learning algorithms XGBoost and RF	84% Accuracy
Muhammad Imran Faisal, Saba Bashir, Zain Sikandar Khan, Farhan Hassan Khan^17^	Ensemble learning method with SVM, GNB, MLP, and NN	90% Accuracy
Proposed methodology	Deep ensemble 2D CNN architecture	95% Accuracy

Table 5 compares the proposed study with the previously presented studies. Knowledge-based Collaborative Deep Learning^11^ obtained the highest accuracy of 94% from the previous studies. An accuracy of 90% was obtained from the Ensemble learning method with SVM, GNB, MLP, and NN^17^. This was the base paper for the study. The study uses the ensemble learning approach for deep learning CNN model for the early identification of Lung cancer from LUNA 16. The proposed study gives an accuracy of 95% with an ensemble learning model considered the highest accuracy in deep learning and ensemble learning algorithms presented to date.

### Gaps and future direction

Our Multilayer CNN is developed by focusing on a 2D Convolution Neural Network. In future work, 3D CNN should be used as 3D can get more spatial information. More data should be gathered to make the model more mature and accurate. An extensive data set will help train the model on a new data set, which will help make the model more accurate. More diverse data will enhance the performance of the model.

There is always room for improvement in any research conducted. There is no final product that has been developed for the detection of any cancer. There has been no international standard developed that will be followed for the detection and prediction of cancers. So, there is always considerable room to increase the accuracy of predictions and detections. More work for detecting and forecasting different cancers will lead to new openings and solutions for detecting cancer in the early stages.

Cancer is a hazardous disease related to a massive number of deaths yearly. Billions of dollars have been spent till now on the research of cancer. Still, no final product has been developed for this purpose. It shows the need for more work to understand the cause and make early predictions. This opens a new opportunity for researchers to develop a system or conduct research that will be very helpful in early cancer detection. If this Is made possible to detect cancer in the very beginning, it can help millions of people out there. There has not been a standard set or final output product which will be used for cancer detections. So, all the researchers should collect current and fresh data and then apply different deep learning and machine learning algorithms to detect and predict cancer. It is essential to use new and existing data, which will help us know whether these Deep Learning and Machine Learning models still give the same accuracy.

## Conclusion

Every year a massive number of deaths are related to cancer which is increasing daily. Billions of dollars have been spent on the research of cancer. It is still an unanswered mystery that needs to be solved. Cancer research is still going on and will be going on and on because no final product has been developed. No specific standards set are used for the detection and prediction of cancer. Cancer research is an open question that needs to get more attention. The latest research on the current data set will open gateways for new research by giving some latest stats and inside stories of what we have achieved till now for the detection and prediction of cancer. It will help to understand some latest causes or signs of cancer.

Many previous studies were presented by the researchers for identifying lung cancer, as discussed in the related work section. The problem of their researchers was low accuracy, lass algorithms, and an inefficient dataset. The proposed study was developed to overcome the loophole of the previous study by using the Deep 2D CNN approach. Three CNN models are used for the proposed study CNN1, CNN2, and CNN3. The results of these three models are deeply explained in Tables 1, 2, and 3. After that, the ensemble 2D approach of deep learning combines all these three deep learning methods. The ensemble deep learning method gives an accuracy of 95%, which is the recorded maximum value of any deep learning algorithm for identifying lung cancer to date. This study shows state-of-the-art results of an ensemble learning approach for identifying lung cancer from the image dataset. In the future, a system may be developed that uses many algorithms in ensemble learning with another extensive and efficient dataset for identifying lung cancer.

## Data Availability

The datasets used and/or analysed during the current study are available from the corresponding author upon reasonable request.
